# Supplementary material for: Individual differences among deep neural network models
Source: Nat Commun. 2020 Nov 12;11:5725. doi: 10.1038/s41467-020-19632-w (PMC7665054; doi:10.1038/s41467-020-19632-w)
Supplement: Supplementary file 1 — Supplementary Information [file 41467_2020_19632_MOESM1_ESM.pdf]

Individual differences among deep neural network models

Johannes Mehrer*[1], Courtney J. Spoerer[1], Nikolaus Kriegeskorte[2], Tim C. Kietzmann[1,3]*

Supplementary information

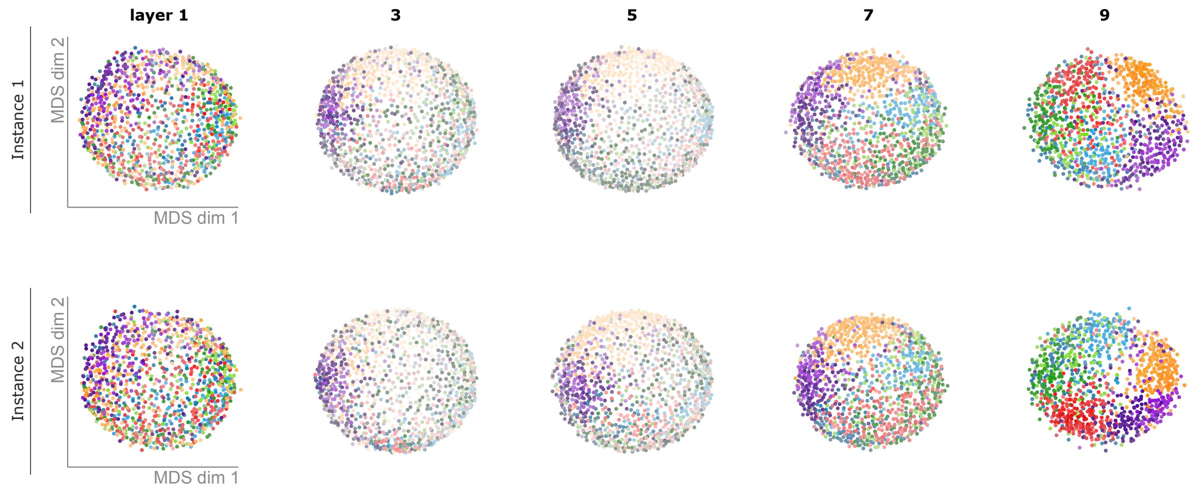

Supplementary Figure 1 | MDS stress for individual datapoint projections. We computed the sum of squared deviations between the original distance estimates and the MDS projection for each datapoint. In the above MDS plot, the color of each point indicates its object category, whereas the color saturation indicates the goodness of the projection (high saturation equals a good fit). Data from a given network instance across all layers and datapoints were normalized to adhere to the same color scale. As can be seen above for two network instances, the reconstruction accuracy of intermediate network layers is worse than early and late layers.

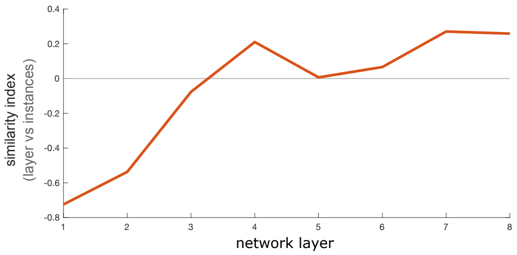

Supplementary Figure 2 | Index comparing representational similarity across instances and layers. We computed the representational similarity across network instances (within a given layer, e.g. the average of the off-diagonal elements in Fig. 3 A cell$_{layer_4,layer_4}$) and subtracted from it the average similarity computed across layers from the same network instance, e.g. diagonal elements in Fig. 3 A in cell$_{layer_4,layer_5}$), standardized by the sum of the two measures. This index demonstrates that starting at layer 4, individual networks are more similar across their adjacent layers than separate instances within a given layer.

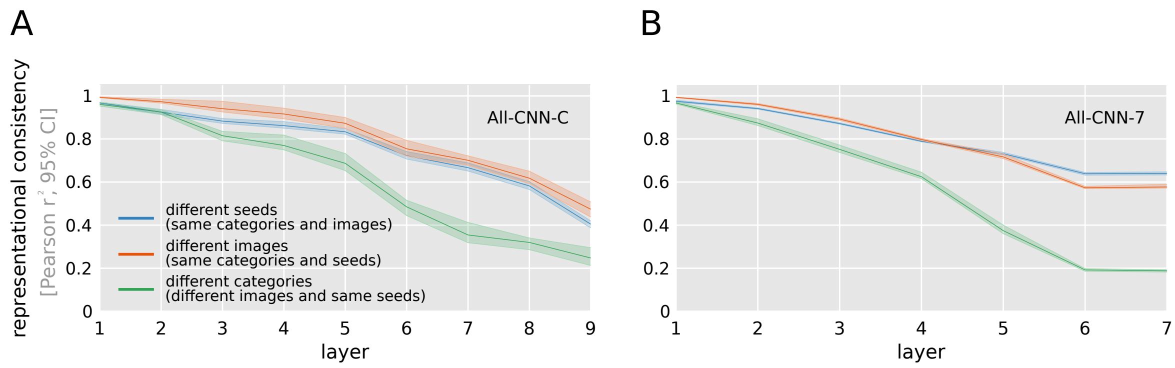

Supplementary Figure 3 | Representational consistency decreases with depth with different input statistics. To better understand the size of the effect in Figure 4, we trained a separate set of networks based on (**A**) All-CNN-C, (**B**) All-CNN-7 while using different images from the same categories but the same seeds (orange), and different categories, different images, and same seeds (green). The minimal intervention of using a different seed for the random weight initialization (shown in blue, data equivalent to Figure 4) affects the internal representations about as much as using a completely different set of training images (10 categories per training set; orange). Please note that part of the larger drop in representational consistency for training with different categories (5 categories per training set; green) can be attributed to training only five categories while computing the RDMs with images from all 10 categories. Data in panels (A) and (B) show average consistency for all pairs of network instances (10 instances,45 pairs), with bootstrapped 95% confidence intervals.

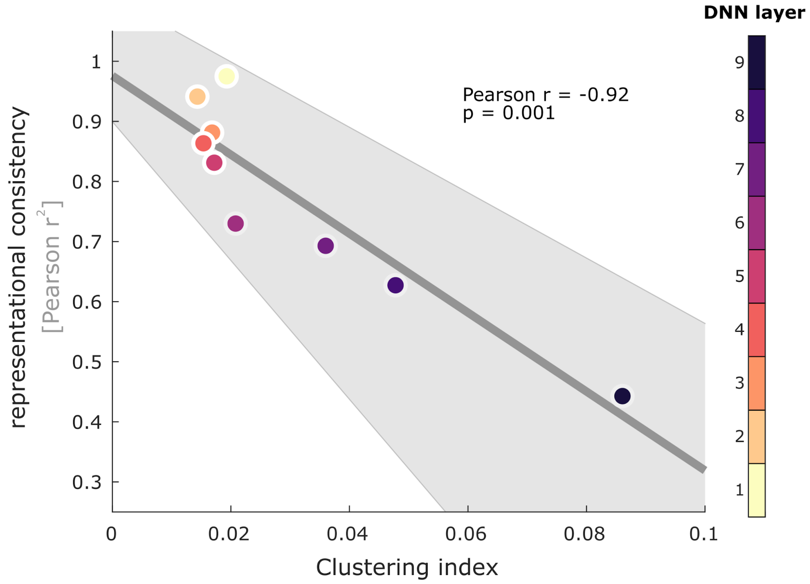

Supplementary Figure 4 | Representational consistency and category clustering are negatively correlated. Optimized for categorization performance, deep neural networks aim to separate images from different categories in the network activation space. Here we show that increasing category separability across All-CNN-C network layers (estimated here by a category clustering index, averaged per layer across network instances) exhibits a negative relationship with representational consistency (consistency values averaged per layer across all network pairs, Pearson *r* = -0.92, *p* = 0.001; robust correlation). That is, individual differences emerge while category clustering increases (95% bootstrapped CIs shown as grey area).

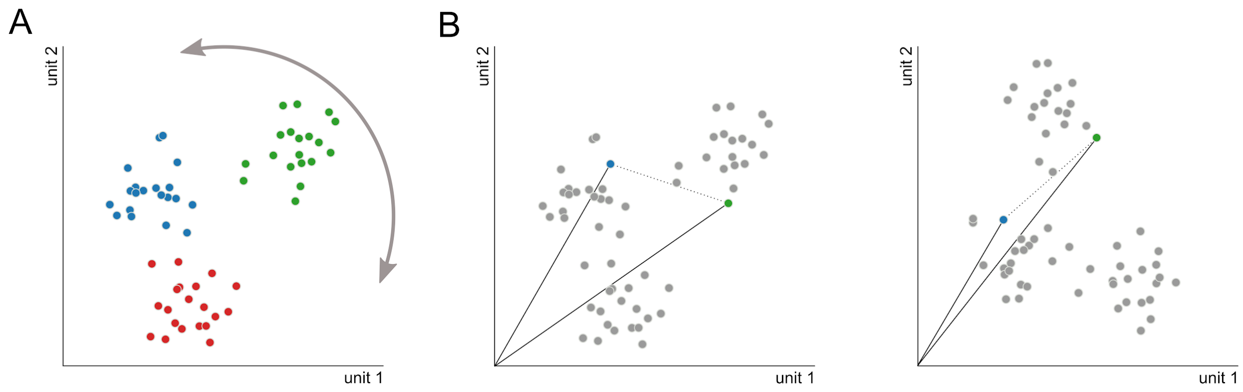

Supplementary Figure 5 | Rotation of a ReLU activation space affects correlation- and cosine-distance estimates. (**A**) Instances of three exemplary object categories (blue, green, red) are rotated in the all-positive (post-ReLU) activation space, here shown as a 2D example. (**B**) When comparing the activation space before (**left panel**) and after the rotation, the angle between pairs of images can differ markedly, thereby leading to lower representational consistency despite an overall stable data arrangement.

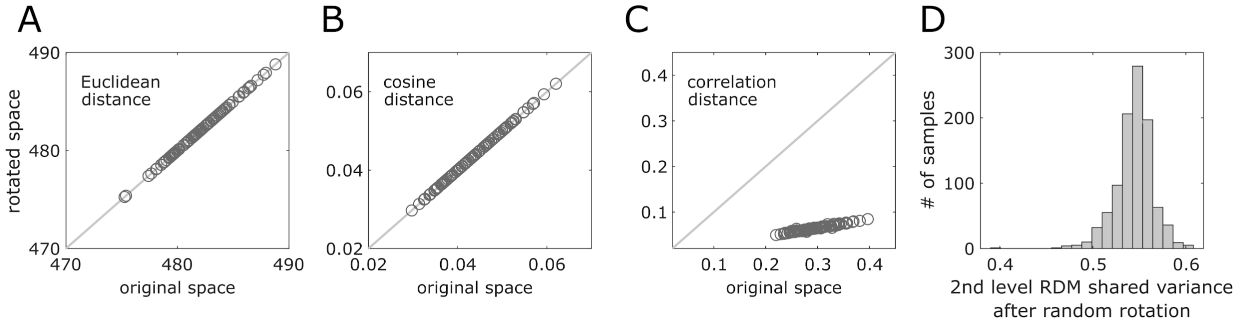

Supplementary Figure 6 | Rotation sensitivity of correlation distance. We computed the distance between two random vectors before and after both vectors were randomly rotated around the origin using the same rotation matrix. This procedure was performed for 100 vector pairs in the above simulation. Rotating both vector pairs does not have an effect when Euclidean or cosine distance is used to compute the vector pair distances (**A, B**). However, when correlation distance is used, rotations around the origin lead to decreased overall distances, and an imperfect correlation between the two distance estimates. Computing a correlation distance involves a projection of the two vectors onto a plane cutting through the origin that is orthogonal to the all-1-vector. This projection differs if the original vectors are rotated. (**C**). Accordingly, when RDMs are based on correlation distance (here based on 10 example responses), rotations around the origin lead to decreased representational consistency, despite the fact that the relative arrangement of datapoints remained identical after the rotation (**D**).

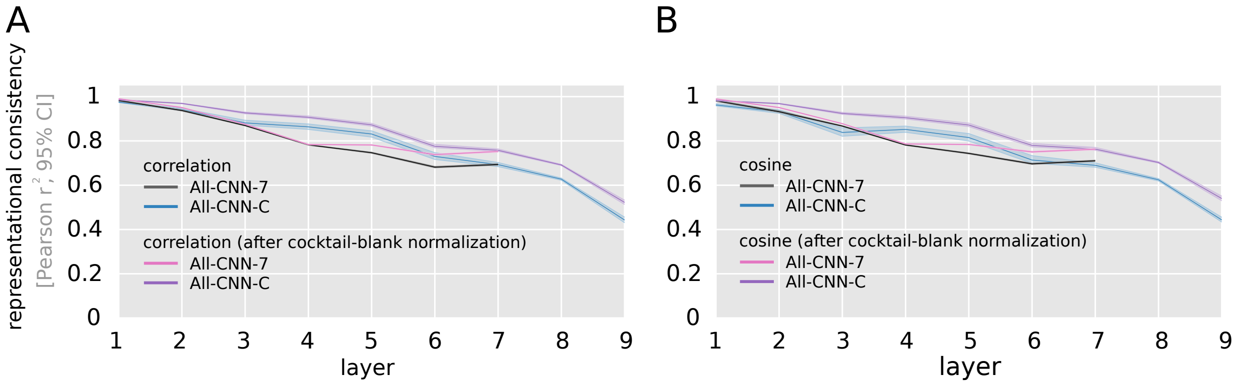

Supplementary Figure 7 | Cocktail blank normalization increases consistency for correlation and cosine distances. Centring the data via cocktail blank normalization increases representational consistency for correlation (**A**) and cosine distance (**B**). Euclidean distance measures are not affected, as the resulting representational geometries are rotationally invariant. Each line shows the average across all pairs of network instances (45 pairs, 10 instances) together with bootstrapped 95% confidence intervals.

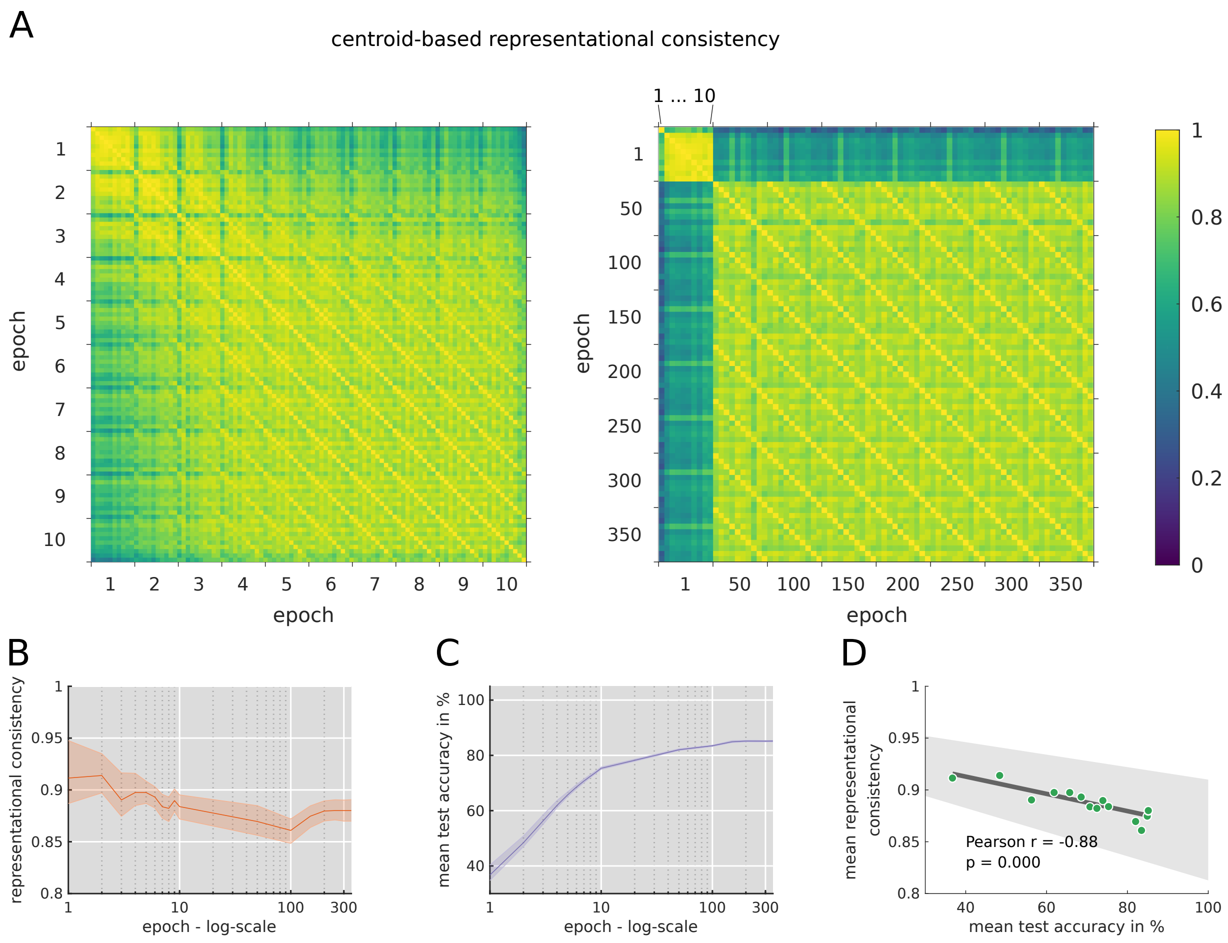

Supplementary Figure 8 | Centroid consistency across training trajectories. Same as Figure 10 of the main manuscript, but computed for RDMs based on category centroids instead of category exemplars. Error bars in panels (B-D) indicate 95% confidence intervals (bootstrapped).

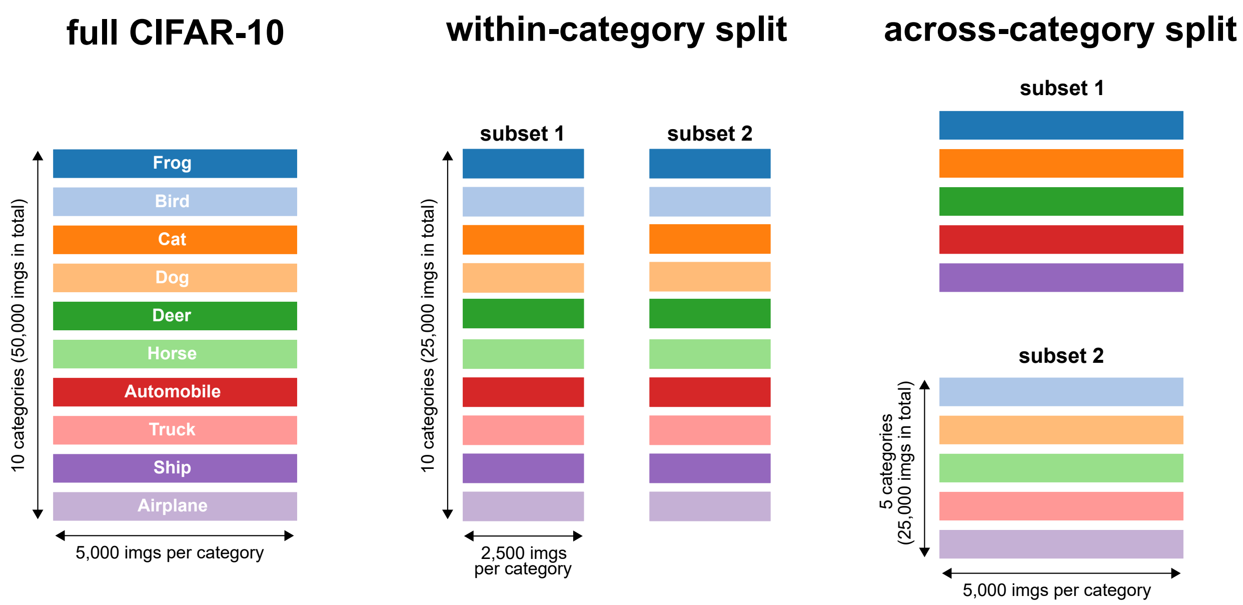

Supplementary Figure 9 | Visualization of the CIFAR-10 training sets used. *Left panel:* The full CIFAR-10 training set consists of 10 categories with 5,000 images each, 50,000 images in total. *Center panel:* the within-category split dataset contains 10 categories with 2,500 images each, 25,000 images in total for each subset. *Right panel*: the across-category split dataset contains 5 categories with 5,000 images each, again 25,000 images in total for each subset. When splitting across categories, the number of animal- and vehicle-categories of the full CIFAR-10 set was equally distributed across the two subsets.

Algorithm 1: DNN representational consistency

```
input  : Image test set, DNN instance 1, DNN instance 2, layer ID
output: representational consistency (shared RDM variance)
for each network instance do
    for each test image do
        extract activation pattern from layer ID;
    for all pairs of test images do
        compute pairwise activation pattern dissimilarity;
        store dissimilarity in RDM upper triangle;
ρ = Pearson(RDM_instance1, RDM_instance2);
consistency = ρ^2;
```

Supplementary Methods | Representational Consistency Pseudocode.
