# Supplementary material for: Individual differences among deep neural network models
Source: Nat Commun. 2020 Nov 12;11:5725. doi: 10.1038/s41467-020-19632-w (PMC7665054; doi:10.1038/s41467-020-19632-w)
Supplement: Supplementary file 4 — Source Data [file 41467_2020_19632_MOESM4_ESM.zip › Source Data/script_01_first_and_second_level_MDS/Corr_toolbox_v2/LIBRA/Academic License LIBRA.pdf]

The following license governs the use of the MATLAB LIBrary for Robust Analysis, (LIBRA) in academic and educational environments. Commercial use requires a commercial license from the Author.

ACADEMIC PUBLIC LICENSE[1]

Copyright© 2005, Mia Hubert

PREAMBLE

This license contains the terms and conditions of using the MATLAB LIBrary for Robust Analysis, hereafter called LIBRA, in a noncommercial settings: at academic institutions for teaching and research use. This license provides the rights similar to the GNU General Public License, yet it retains the possibility for LIBRA authors to financially support the development by selling commercial licenses. In fact, if you intend to use LIBRA in a "for-profit" environment, where research is conducted to develop or enhance a product, or it is used in a commercial service offering, you need to obtain a commercial license for LIBRA which is available from the Author.

What are the rights given to noncommercial users? Similarly to GPL, you have the right to use the software, to distribute copies, to receive source code, to change the software and distribute your modifications or the modified software. Also similarly to the GPL, if you distribute verbatim or modified copies of this software, they must be distributed under this license.

By modeling the GPL, this license guarantees that you're safe when using LIBRA in your work, for teaching or research. This license guarantees that LIBRA will remain available free of charge for nonprofit use.

The precise terms and conditions for using, copying, distribution and modification follow.

[1] After the model of Andras Vargas for OMNeT++, http://www.omnetpp.org/external/license.php

TERMS AND CONDITIONS FOR USE, COPYING, DISTRIBUTION AND MODIFICATION

"*Program*" means a copy of LIBRA, which is said to be distributed under this Academic Public License.

"*Work based on the Program*" means either the Program or any derivative work under copyright law: that is to say, a work containing the Program or a portion of it, either verbatim or with modifications and/or translated into another language. (Hereinafter, translation is included without limitation in the term "modification".)

"*Using the Program*" means any act of creating executables that contain or directly use libraries that are part of the Program, running any of the tools that are part of the Program, or creating works based on the Program.

Each licensee is addressed as "*you*".

1. Permission is hereby granted to use the Program free of charge for any noncommercial purpose, including teaching and research at universities, colleges and other educational institutions, research at non-profit research institutions, and personal non-profit purposes. For using the Program for commercial purposes, including but not restricted to consulting activities and design of commercial hardware or software networking products, you have to contact the Author for an appropriate license. Permission is also granted to use the Program for a reasonably limited period of time for the purpose of evaluating its usefulness for a particular purpose.

2. You may copy and distribute verbatim copies of the Program's source code as you receive it, in any medium, provided that you conspicuously and appropriately publish on each copy an appropriate copyright notice and disclaimer of warranty; keep intact all the notices that refer to this License and to the absence of any warranty; and give any other recipients of the Program a copy of this License along with the Program.

3. You may modify your copy or copies of the Program or any portion of it, thus forming a work based on the Program, and copy and distribute such modifications or work under the terms of Section 2 above, provided that you also meet all of these conditions:

a) You must cause the modified files to carry prominent notices stating that you changed the files and the date of any change.

b) You must cause any work that you distribute or publish, that in whole or in part contains or is derived from the Program or any part thereof, to be licensed as a whole at no charge to all third parties under the terms of this License.

These requirements apply to the modified work as a whole. If identifiable sections of that work are not derived from the Program, and can be reasonably considered independent and separate works in themselves, then this License, and its terms, do not apply to those sections when you distribute them as separate works. But when you distribute the same sections as part of a whole which is a work based on the Program, the distribution of the whole must be on the terms of this License, whose regulations for other licensees extend to the entire whole, and thus to each and every part regardless of who wrote it. (If the same, independent sections are distributed as part of a package that is otherwise reliant on, or is based on the Program, then the distribution of the whole package, including but not restricted to the independent section, must be on the unmodified terms of this License, regardless of who the author of the included sections was.)

Thus, it is not the intent of this section to claim rights or contest your rights to work written entirely by you; rather, the intent is to exercise the right to control the distribution of derivative or collective works based or reliant on the Program.

In addition, mere aggregation of another work not based on the Program with the Program (or with a work based on the Program) on a volume of storage or distribution medium does not bring the other work under the scope of this License.

4. You may copy and distribute the Program (or a work based on it, under Section 3) in object code or executable form under the terms of Sections 2 and 3 above provided that you also do one of the following:

a) Accompany it with the complete corresponding machine-readable source code, which must be distributed under the terms of Sections 2 and 3 above on a medium customarily used for software interchange; or,

b) Accompany it with the information you received as to the offer to distribute corresponding source code. (This alternative is allowed only for noncommercial distribution and only if you received the program in object code or executable form with such an offer, in accord with Subsection b) above.)

The source code for a work means the preferred form of the work for making modifications to it. For an executable work, complete source code means all the source code for all modules it contains, plus any associated interface definition files, plus the scripts used to control compilation and installation of the executable. However, as a special exception, the source code distributed need not include anything that is normally distributed (in either source or binary form) with the major components (compiler, kernel, and so on) of the operating system on which the executable runs, unless that component itself accompanies the executable.

If distribution of executable or object code is made by offering access to copy from a designated place, then offering equivalent access to copy the source code from the same place counts as distribution of the source code, even though third parties are not compelled to copy the source along with the object code.

5. You may not copy, modify, sublicense, or distribute the Program except as expressly provided under this License. Any attempt otherwise to copy, modify, sublicense or distribute the Program is void, and will automatically terminate your rights under this License. However, parties who have received copies, or rights, from you under this License will not have their licenses terminated as long as such parties remain in full compliance.

6. You are not required to accept this License, since you have not signed it. Nothing else grants you permission to modify or distribute the Program or its derivative works; law prohibits these actions if you do not accept this License. Therefore, by modifying or distributing the Program (or any work based on the Program), you indicate your acceptance of this License and all its terms and conditions for copying, distributing or modifying the Program or works based on it, to do so.

7. Each time you redistribute the Program (or any work based on the Program), the recipient automatically receives a license from the original licensor to copy, distribute or modify the Program subject to these terms and conditions. You may not impose any further restrictions on the recipients' exercise of the rights granted herein. You are not responsible for enforcing compliance by third parties to this License.

8. If, as a consequence of a court judgment or allegation of patent infringement or for any other reason (not limited to patent issues), conditions are imposed on you (whether by court order, agreement or otherwise) that contradict the conditions of this License, they do not excuse you from the conditions of this License. If you cannot distribute so as to satisfy simultaneously your obligations under this License and any other pertinent obligations, then as a consequence you may not distribute the Program at all. For example, if a patent license would not permit royalty-free redistribution of the Program by all those who receive copies directly or indirectly through you, then the only way you could satisfy both it and this License would be to refrain entirely from distribution of the Program.

If any portion of this section is held invalid or unenforceable under any particular circumstance, the balance of the section is intended to apply and the section as a whole is intended to apply in other circumstances.

9. If the distribution and/or use of the Program are restricted in certain countries either by patents or by copyrighted interfaces, the original copyright holder who places the Program under this License may add an explicit geographical distribution limitation excluding those countries, so that distribution is permitted only in or among countries not thus excluded. In such case, this License incorporates the limitation as if written in the body of this License.

10. If you wish to incorporate parts of the Program into other free programs whose distribution conditions are different, write to the Author to ask for permission.

NO WARRANTY

10. BECAUSE THE PROGRAM IS LICENSED FREE OF CHARGE, THERE IS NO WARRANTY FOR THE PROGRAM, TO THE EXTENT PERMITTED BY APPLICABLE LAW. EXCEPT WHEN OTHERWISE STATED IN WRITING THE COPYRIGHT HOLDERS AND/OR OTHER PARTIES PROVIDE THE PROGRAM "AS IS" WITHOUT WARRANTY OF ANY KIND, EITHER EXPRESSED OR IMPLIED, INCLUDING, BUT NOT LIMITED TO, THE IMPLIED WARRANTIES OF MERCHANTABILITY AND FITNESS FOR A PARTICULAR PURPOSE. THE ENTIRE RISK AS TO THE QUALITY AND PERFORMANCE OF THE PROGRAM IS WITH YOU. SHOULD THE PROGRAM PROVE DEFECTIVE, YOU ASSUME THE COST OF ALL NECESSARY SERVICING, REPAIR OR CORRECTION.

11. IN NO EVENT UNLESS REQUIRED BY APPLICABLE LAW OR AGREED ON IN WRITING WILL ANY COPYRIGHT HOLDER, OR ANY OTHER PARTY WHO MAY MODIFY AND/OR REDISTRIBUTE THE PROGRAM AS PERMITTED ABOVE, BE LIABLE TO YOU FOR DAMAGES, INCLUDING ANY GENERAL, SPECIAL, INCIDENTAL OR CONSEQUENTIAL DAMAGES ARISING OUT OF THE USE OR INABILITY TO USE THE PROGRAM INCLUDING BUT NOT LIMITED TO LOSS OF DATA OR DATA BEING RENDERED INACCURATE OR LOSSES SUSTAINED BY YOU OR THIRD PARTIES OR A FAILURE OF THE PROGRAM TO OPERATE WITH ANY OTHER PROGRAMS), EVEN IF SUCH HOLDER OR OTHER PARTY HAS BEEN ADVISED OF THE POSSIBILITY OF SUCH DAMAGES.

END OF TERMS AND CONDITIONS
