# Supplementary material for: Individual differences among deep neural network models
Source: Nat Commun. 2020 Nov 12;11:5725. doi: 10.1038/s41467-020-19632-w (PMC7665054; doi:10.1038/s41467-020-19632-w)
Supplement: Supplementary file 4 — Source Data [file 41467_2020_19632_MOESM4_ESM.zip › Source Data/script_01_first_and_second_level_MDS/Corr_toolbox_v2/manual.pdf]

Robust Correlation toolbox Manual

The toolbox allows performing robust correlation analyses along with various assumption checks and data visualization.

If you use the toolbox for your research, please cite:

Pernet, C.R., Wilcox, R. & Rousselet, G.A. (2013). Robust correlation analyses: false positive and power validation using a new open source Matlab toolbox. *Front. in Psychology, 3, 606. doi: 10.3389/fpsyg.2012.00606*

If you use the skipped-correlation, which depends on an estimation of the robust centre of the data, then please also cite:

Rousseeuw, P.J. (1984), "Least Median of Squares Regression," Journal of the American Statistical Association, Vol. 79, pp. 871-881.

Rousseeuw, P.J. and Van Driessen, K. (1999), "A Fast Algorithm for the Minimum Covariance Determinant Estimator," Technometrics, 41, pp. 212-223.

Verboten, S., & Hubert, M. (2005). LIBRA: a MATLAB Library for Robust Analysis, *Chemometrics and Intelligent Laboratory Systems*(75), 127–136.

If you report the test of multivariate normality, please cite:

Trujillo-Ortiz, A., R. Hernandez-Walls, K. Barba-Rojo and L. Cupul-Magana. (2007). HZmvntest:Henze-Zirkler's Multivariate Normality Test. A MATLAB file.

A description of the usefulness of such methods can also be found in

Rousselet, G. A., and Pernet, C. R. (2012). Improving standards in brain-behavior correlation analyses. Front. Hum. Neurosci. 6:119.

Table of Contents

1 – Before starting..
2 – Importing data in Matlab...
3 – One-step analysis between 2 variables ....
4 - Calling specific function..
5 – Multiple testing solutions ....
6 - References...

1 – Before starting

If you are not familiar with Matlab, all you need to do to get the toolbox to work is to set the path. That means that you tell Matlab where the toolbox is located. The easiest way to do so is to click at the top of the Matlab window on *File →Set Path*, then click on *Add with Subfolders* and select the Corr_toolbox.

2 – Importing data in Matlab

For a Matlab novice it might seem complicated to import data. This is however as easy as with any other software. Here is how to load the Anscombe's quartet data, which can be found in the toolbox folder as an Excel file (Anscombe.xls), a text file (Anscombe.txt) and a Matlab file (Anscombe.mat).

Import excel file data: in Malab's 'Current Folder' drop down menu, navigate to the toolbox folder, open the folder, then double click on Anscombe.xls. The import wizard is now open and the data are already selected (figure 1). At the top left of the wizard, you have the choice about how to import: select 'Column vectors' and then click on 'Import'. You should now have in the 'Workspace' 8 variables called X1, X2, X3, X4, Y1, Y2, Y3, Y4.

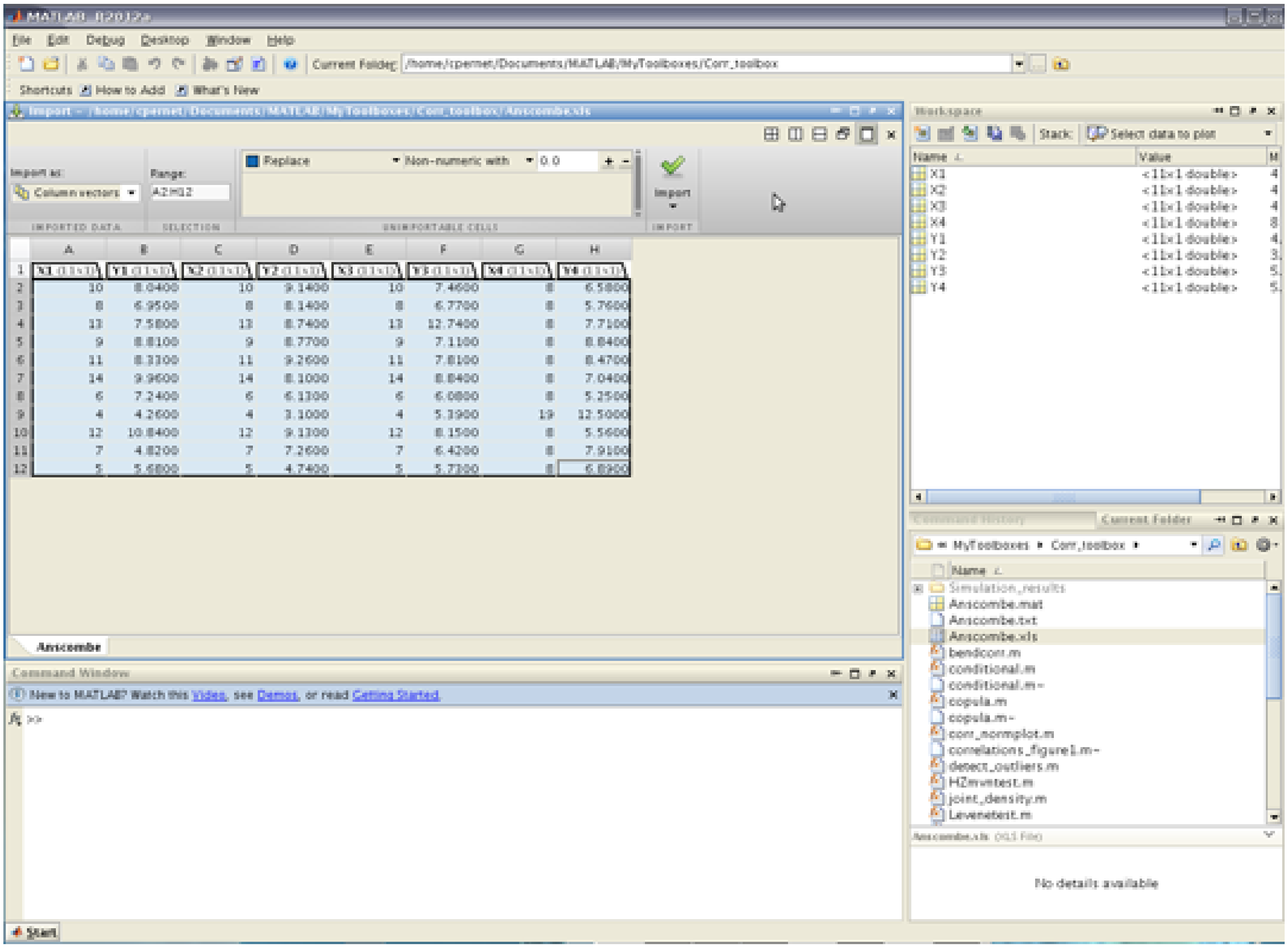

Figure 1. Matlab import wizard for the excel file 'Anscombe.xls'.

Import text file data: in Malab's 'Current Folder' drop down menu, navigate to the toolbox folder, open the folder, then right click on Anscombe.txt, and select Import Data. The import wizard is now open (figure 2). Click next and you're finished. You should now have all the variables loaded in the 'Workspace' as a single matrix called Anscombe.

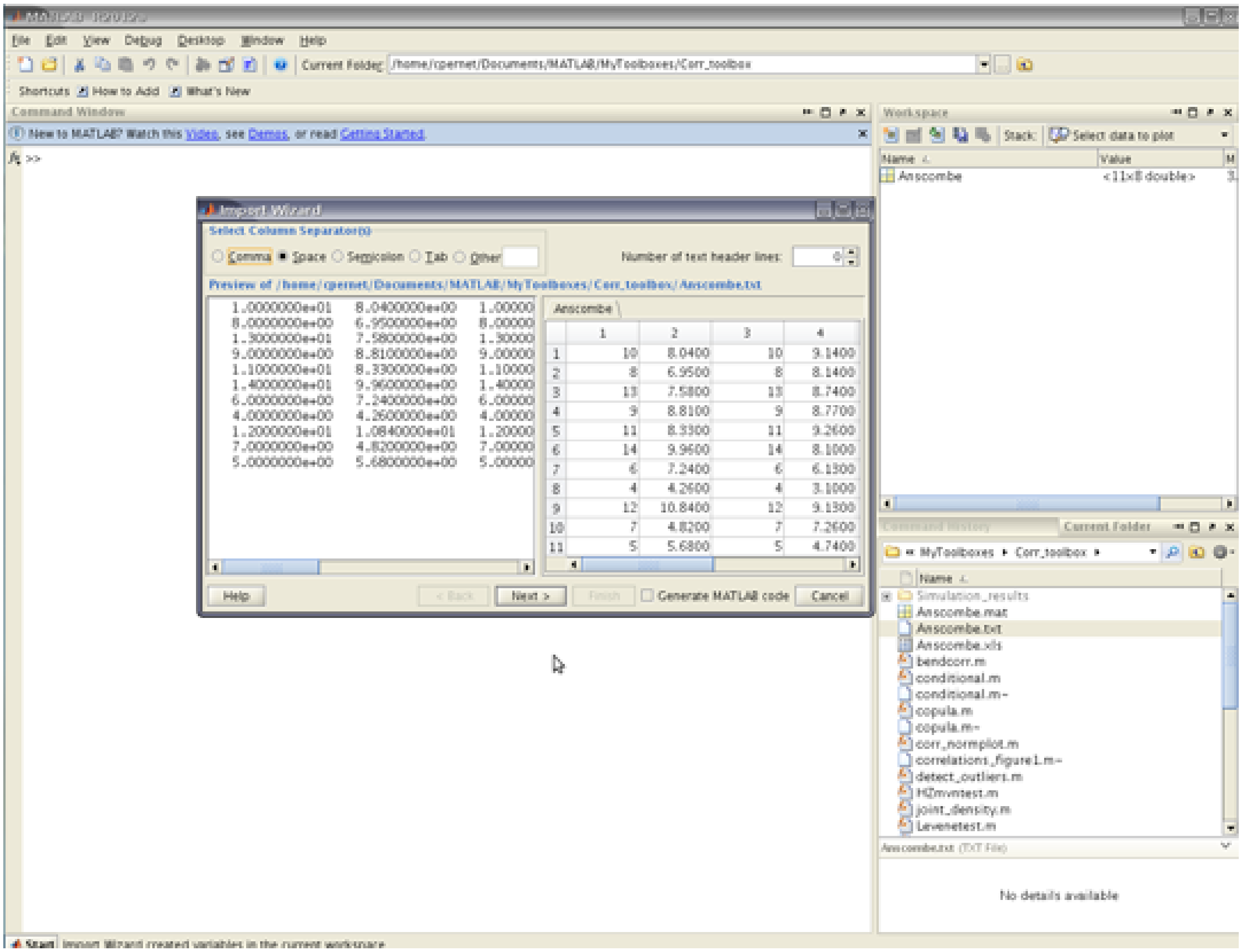

Figure 2. Matlab import wizard for the text file 'Anscombe.txt'.

Load data already in Matlab format: once the data have been imported, it is easier to save them in Matlab format to be used later (type *help save* in the command window). In that case the data can be accessed by double clicking on Anscombe.mat from the Malab 'Current Folder' window.

3 – One-step analysis between 2 variables

When only 2 variables are tested, all the tests and plots available in the toolbox can be performed in one step, by calling the *robust_correlation.m* function. Load the data Anscombe.mat (section 2) and in the Matlab command window, type:

```
>> correlation_results = robust_correlation(Anscombe(:,1),Anscombe(:,2))
```

The function performs the following operations:

(1) plots the data with (i) a scatter plot, (ii) the marginal (normalized) histograms with the corresponding Gaussian curves, and (iii) the bivariate histogram (*corr_normplot.m*);
(2) plots the joint density as a mesh and its isocontour (*joint_density.m*).
(3) tests bivariate normality (*Hzmvntest.m*);
(4) tests heteroscedasticity (*variance_honogeneity.m*);
(5) looks for outliers (*detect_outliers.m*);
(6) performs 4 types of correlations (*Pearson.m, Spearman.m, bendcorr.m, skipped-correlation.m*).

As the different tests are performed, outputs appear in the Matlab command window and as graphics (figures 3 and 4). In addition, there is now a structure called *correlation_results* in the workspace: it contains all the results. For instance, typing `correlation_results.Pearson` returns the r, t, and p values, the bootstrap confidence intervals and a message telling you if it is significant or not, based on the bootstrap confidence intervals.

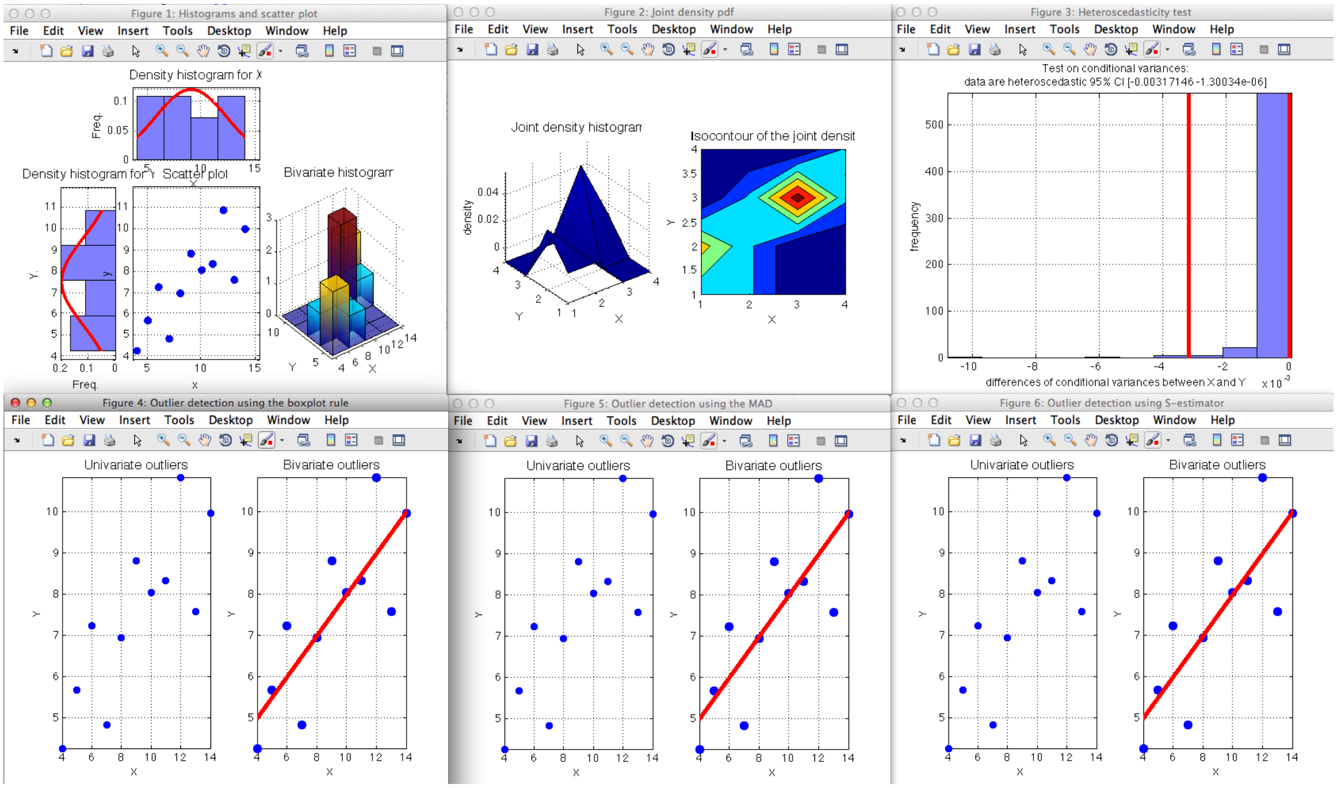

Figure 3. Graphical outputs returned by the correlation toolbox for the 1st Anscombe's quartet. Top left (Figure 1) = scatter plots and histograms. Top middle (Figure 2) = mesh and isocontours of the joint density. Top right (Figure 3) = histogram of the differences in the conditional variances of bootstrapped data. Bottom row (Figures 4-6) = univariate and bivariate outliers detected using the boxplot rule, the MAD-median rule, or an S-estimator.

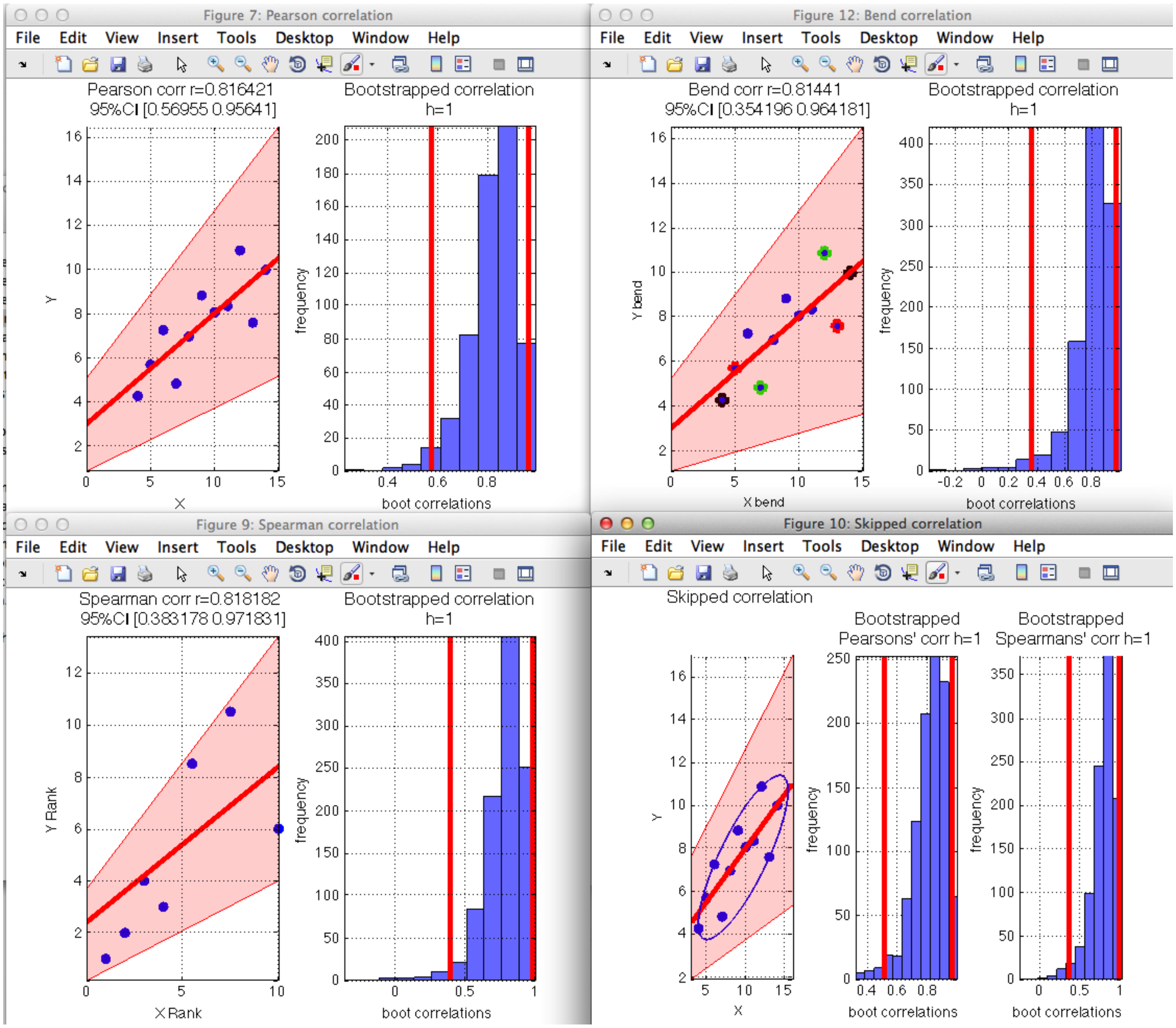

Figure 4. Graphical outputs returned by the correlation toolbox for the 1st Anscombe's quartet. Top left (Figure 7) = Pearson correlation with 95% CI and the histogram of correlations for bootstrapped data. Top right (Figure 8) = 20% bend correlation with 95% CI and the histogram of correlations for bootstrapped data. Bottom left (Figure 9) = Spearman correlation with 95% CI and the histogram of correlations for bootstrapped data. Bottom right (Figure 10) = Skipped correlation with 95% CI and the histograms of Pearson's and Spearman's correlations for bootstrapped data.

4 - Calling specific functions

Data visualization

To get the scatter plot with histograms type:
```
corr_normplot(Anscombe(:,1), Anscombe(:,2));
```

To get the joint density type:
```
density = joint_density(Anscombe(:,1), Anscombe(:,2));
```

It will not only plots the joint density, but also returns it as output variable. For instance in the above example you will get density =

0.0221	0.0221	0	0
0.0442	0.0221	0	0
0	0.0221	0.0663	0.0221
0	0	0	0.0221

It is also possible to only plot the isocontours by typing
```
density = joint_density(Anscombe(:,1), Anscombe(:,2),0);
```

Assumption checking

To test for multivariate normality, type:
```
HZmvntest([Anscombe(:,1), Anscombe(:,2)]);
```
Note the square brackets [ ] as the function needs a single matrix as input.

Variance homogeneity can be tested by typing:
```
[h,CI] = variance_homogeneity(Anscombe(:,1),Anscombe(:,2));
```

h=1 if data have different variances, and h=0 if data have the same variances. CI is the percentile bootstrap 95% confidence interval of the difference between variances.

By default the function normalizes the data and uses conditional variances (see conditional.m). It is however possible to force the function to use the original variances by typing:
```
[h,CI] = variance_homogeneity(Anscombe(:,1),Anscombe(:,2),0);
```

To detect outliers using robust estimators type:
```
outliers = detect_outliers(Anscombe(:,1),Anscombe(:,2));
```

The output o*utliers* is a structure in which the field *outliers.univariate.X* contains indices of univariate outliers in X, *outliers.univariate.Y* contains indices of univariate outliers in Y, and *outliers.bivariate* returns bivariate outliers. In each case, outliers are marked by 1s, and other points as 0s.

Correlations

Pearson
`[r,t,p] = Pearson(Anscombe(:,1),Anscombe(:,2))`
returns the correlation (r) value along with the t and p values and makes a plot of the data with a line representing the best linear fit.

`[r,t,p] = Pearson(Anscombe(:,1),Anscombe(:,2),0)`
does the same thing but without making a figure.

`[r,t,p,hboot,CI] = Pearson(Anscombe(:,1),Anscombe(:,2),1,10/100)`
returns a decision about significance (hboot) based on the bootstrap confidence intervals (CI) at the desired type 1 level (here 10% – default is 5%). Note the difference in graphical outputs: because the bootstrap was used, CI and the histogram of bootstrapped correlations are also plotted.

Spearman
`[r,t,p] = Spearman(Anscombe(:,1),Anscombe(:,2))`
returns the correlation (r) value along with the t and p values and makes a plot of the data with a line representing the best linear fit. Note the difference with Pearson: with Sperman the scatter plot is for the ranked data.

`[r,t,p] = Spearman(Anscombe(:,1),Anscombe(:,2),0)`
does the same thing but without making a figure.

`[r,t,p,hboot,CI] = Spearman(Anscombe(:,1),Anscombe(:,2),1,10/100)`
returns a decision about significance (hboot) based on the bootstrap confidence intervals (CI) at the desired type 1 level (here 10% - default is 5%). Note the difference in graphical outputs: because the bootstrap was used, CI and the histogram of bootstrapped correlations are also plotted.

Percentage bend correlation
`[r,t,p] = bendcorr(Anscombe(:,1),Anscombe(:,2))`
returns the correlation (r) value along with the t and p values and makes a plot of the data with a line representing the best linear fit. In the graphical output different colors are used to indicate which data points were weighted down: red for data in X, green for data in Y and black for data both in X and in Y). The percentage bend correlation is not an estimate of Pearson's correlation – it is however a measure of the linear relationship between X and Y.

`[r,t,p,hboot,CI] = bendcorr(Anscombe(:,1),Anscombe(:,2),0,40)`
returns a decision about significance (hboot) based on the bootstrap 95% confidence intervals (CI) – additional arguments indicate not to plot the data (0) and use 40% bending rather than the default 20%.

Skipped-correlation
`[r,t,h] = skipped_correlation(Anscombe(:,1),Anscombe(:,2))`
`[r,t,h] = skipped_correlation(Anscombe(:,1),Anscombe(:,2),0)`
returns the correlation (r) values, along with the t values of Pearson and

Spearman tests performed on data after removing bivariate outliers. No p value is computed, but the T value is thresholded such that a decision h can be returned for alpha = 5%. This is performed with adjustments related to the sample size to maintain the type 1 error rate at the nominal level. By default, the function also makes a plot of the data with an ellipse containing non outlying data and a line representing the best linear fit to the remaining data points. It is essential to understand that outlier removal is based on normality, since we use the MCD. Spearman is computed on the same data as Pearson, and thus is not optimized for non-linear relationships.

```
[r,t,h,outid,hboot,CI] = skipped_correlation(Anscombe(:,1),Anscombe(:,2))
```

also returns outlier data in outid, which is the same as using *outlier_detect*. It also adds 95% bootstrap confidence intervals (CI) and a decision about statistical significance (hboot).

5 – Multiple testing solutions

If you perform multiple tests, you increase the chances to make a false positive error. This is only true for families of tests that are related to each other, for instance if you try to look at the relationship between age and scores in different cognitive tests, all obtained from the same subjects. If you do 2 tests on unrelated pairs of variables, then you do not need to control for multiple tests. If on the other hand variables are related, the risk of false positives increases with the number of tests. We illustrate how to correct for multiple tests using data from the Anscombe's quartet, which form a family of tests.

Pearson and Spearman

```
[r,t,p,hboot,CI] = Pearson(Anscombe(:,1),Anscombe(:,[2 4 6]))
[r,t,p,hboot,CI] = Pearson(Anscombe(:,[1 3 5]),Anscombe(:,[2 4 6]))
[r,t,p,hboot,CI] = Spearman(Anscombe(:,1),Anscombe(:,[2 4 6]))
[r,t,p,hboot,CI] = Spearman(Anscombe(:,[1 3 5]),Anscombe(:,[2 4 6]))
```

Here either one vector and a matrix or two matrices of data were input, and correlations are always computed column-wise (the function repeats the vector Anscombe(:,1) for 1 vector and a matrix as inputs).

Multiple comparison correction is performed using a Bonferroni correction: for 3 tests, only p values below 1.67% (5% / 3) are considered significant, and confidence intervals are adjusted to 98.33% accordingly.

Percentage bend correlation

```
[r,t,p,hboot,CI,H,pH] = bendcorr(Anscombe(:,1),Anscombe(:,[2 4 6]))
[r,t,p,hboot,CI,H,pH] = bendcorr(Anscombe(:,[1 3 5]),Anscombe(:,[2 4
6]))
```

As for Pearson and Spearman, a vector and a matrix or 2 matrices can be input and correlations are computed column-wise. Also, hboot and CI are adjusted

using a Bonferroni correction.

In output, H and pH can also be added to compute an omnibus test of independence between all pairs, by testing if the correlation matrix is equal to the identify matrix (0 everywhere expect 1s in the diagonal). H is the measure of association between all pairs (as r is the measure of association between two variables for 1 pair) and pH is the associated p value.

Skipped-correlation

```
[r,t,h,outid,hboot,CI] = skipped_correlation(Anscombe(:,1
),Anscombe(:,[2 4 6]))
```

A vector and a matrix are used as inputs, and h returns the significance with a n adjustment for multiple comparisons

```
[r,t,h,outid,hboot,CI] = skipped_correlation(Anscombe(:,[1 3 5]
),Anscombe(:,[2 4 6]))
```

Two matrices are used as inputs, and h returns the significance with a adjustment for multiple comparisons, testing that all correlations are 0.

In both cases, r and t are only computed for Spearman, because only Spearman provides a good type 1 error rate in the context of multiple comparisons – hboot and CI are adjusted using a Bonferroni correction.

6 - References

Henze, N. and Zirkler, B. (1990), A Class of Invariant Consistent Tests for Multivariate Normality. Communication in Statistics – Theory and Methods, 19(10): 3595-3618.

Pernet, C.R., Wilcox, R. & Rousselet, G.A. (2013). Robust correlation analyses: false positive and power validation using a new open source Matlab toolbox. *Front. in Psychology, 3, 606. doi: 10.3389/fpsyg.2012.00606*

Rousseeuw, P.J. (1984), "Least Median of Squares Regression," Journal of the American Statistical Association, Vol. 79, pp. 871-881.

Rousseeuw, P.J. and Van Driessen, K. (1999), "A Fast Algorithm for the Minimum Covariance Determinant Estimator," Technometrics, 41, pp. 212-223.

Rousselet GA and Pernet CR (2012) Improving standards in brain-behavior correlation analyses. *Front. Hum. Neurosci.* **6**:119. doi: 10.3389/fnhum.2012.00119

Trujillo-Ortiz, A., R. Hernandez-Walls, K. Barba-Rojo and L. Cupul-Magana. (2007). HZmvntest:Henze-Zirkler's Multivariate Normality Test. A MATLAB file. http://www.mathworks.com/matlabcentral/fileexchange/loadFile.do?objectId=17931

Verboten, S., & Hubert, M. (2005). LIBRA: a MATLAB Library for Robust Analysis, *Chemometrics and Intelligent Laboratory Systems*(75), 127–136.

Wilcox, R. (2012). Introduction to robust estimation and hypothesis testing. 3rd Edition. Elsevier, *Academic Press, Oxford, UK.*
