# Supplementary figures and images for: Individual differences among deep neural network models
Source: Nat Commun. 2020 Nov 12;11:5725. doi: 10.1038/s41467-020-19632-w (PMC7665054; doi:10.1038/s41467-020-19632-w)

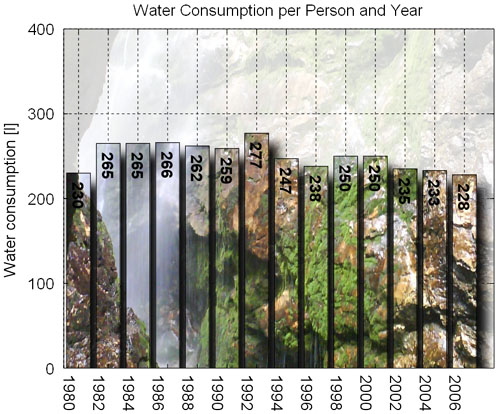

Supplement: Supplementary file 4 — Source Data [file 41467_2020_19632_MOESM4_ESM.zip › Source Data/script_01_first_and_second_level_MDS/plot2svg/demo_svg_water.jpg]

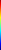

Supplement: Supplementary file 4 — Source Data [file 41467_2020_19632_MOESM4_ESM.zip › Source Data/script_01_first_and_second_level_MDS/plot2svg/gradient.png]

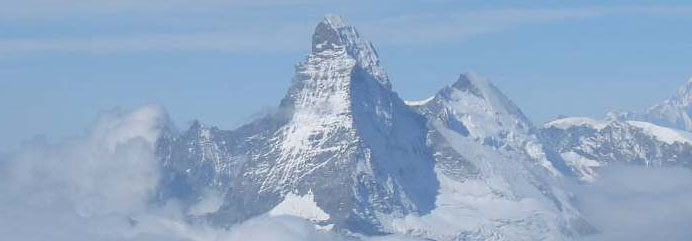

Supplement: Supplementary file 4 — Source Data [file 41467_2020_19632_MOESM4_ESM.zip › Source Data/script_01_first_and_second_level_MDS/plot2svg/matterhorn_small.jpg]

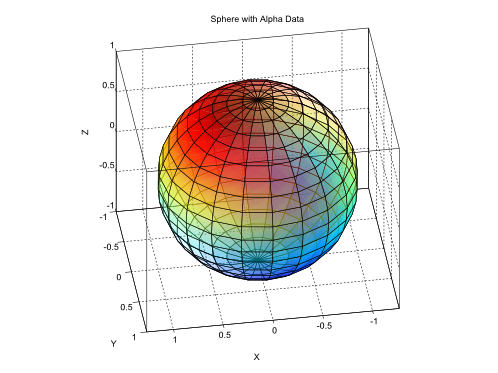

Supplement: Supplementary file 4 — Source Data [file 41467_2020_19632_MOESM4_ESM.zip › Source Data/script_01_first_and_second_level_MDS/plot2svg/sphere.png]

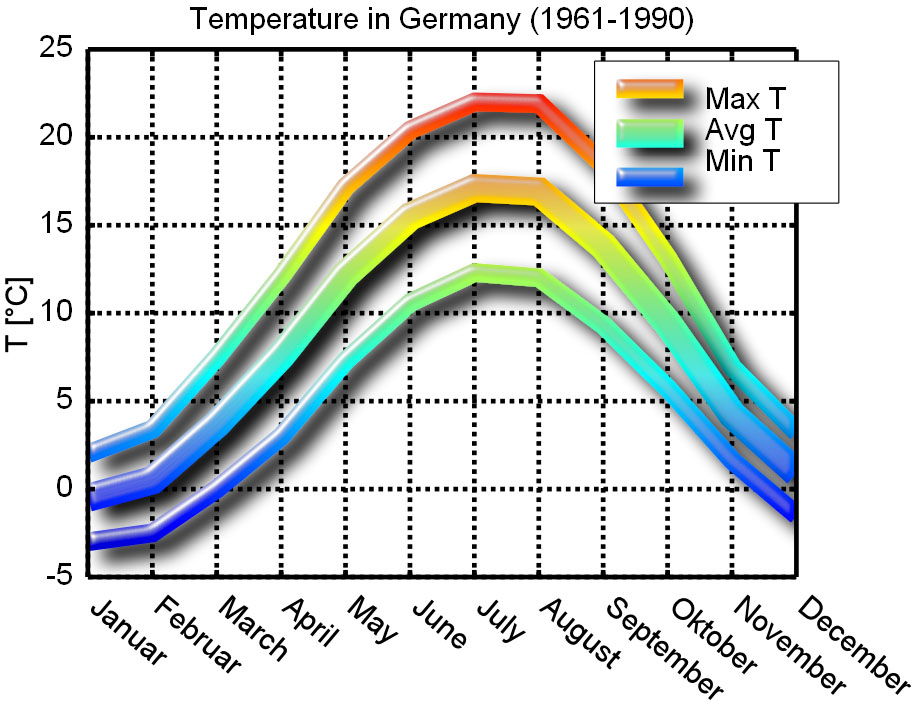

Supplement: Supplementary file 4 — Source Data [file 41467_2020_19632_MOESM4_ESM.zip › Source Data/script_01_first_and_second_level_MDS/plot2svg/temperature_perfect.jpg]

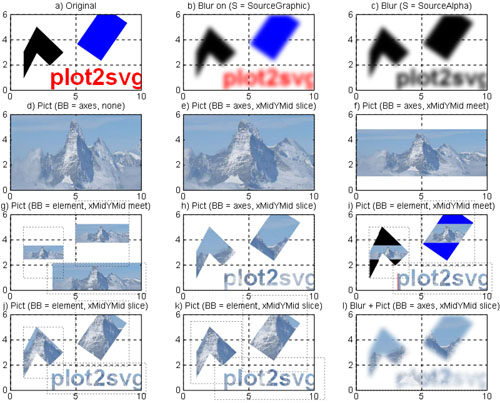

Supplement: Supplementary file 4 — Source Data [file 41467_2020_19632_MOESM4_ESM.zip › Source Data/script_01_first_and_second_level_MDS/plot2svg/tutorial_filters.jpg]

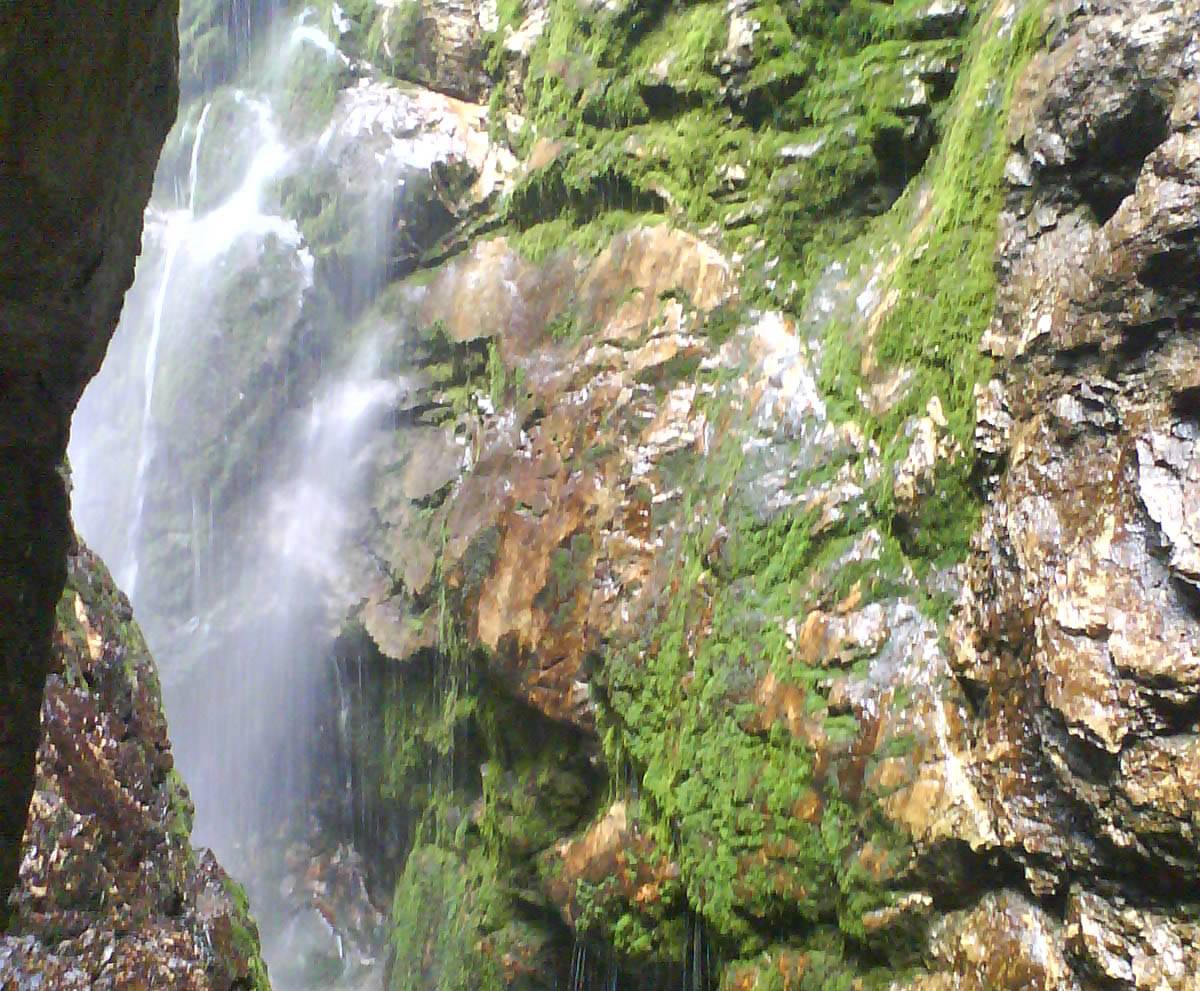

Supplement: Supplementary file 4 — Source Data [file 41467_2020_19632_MOESM4_ESM.zip › Source Data/script_01_first_and_second_level_MDS/plot2svg/water_stones.jpg]

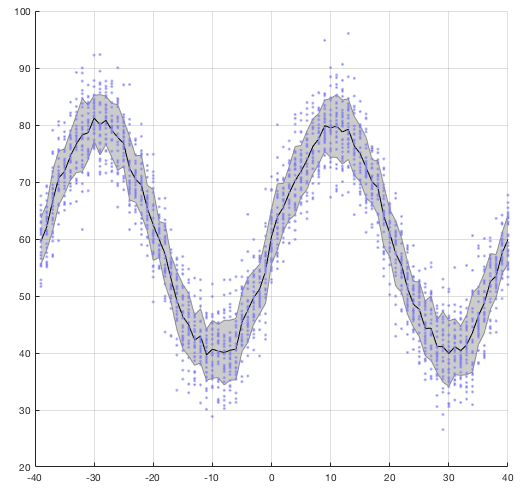

Supplement: Supplementary file 4 — Source Data [file 41467_2020_19632_MOESM4_ESM.zip › Source Data/script_02_consistency_decreases_with_layer_depth/raacampbell-shadedErrorBar/exampleImages/basic_with_overlay.png]

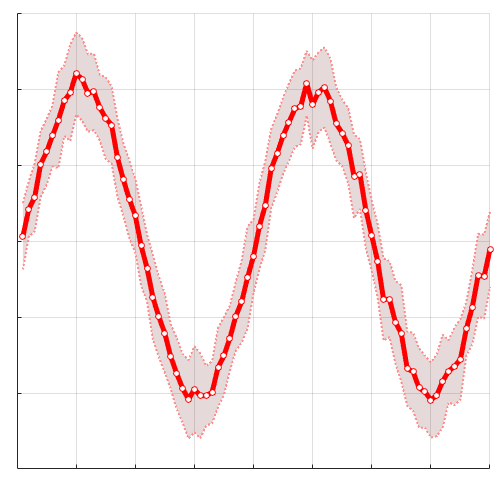

Supplement: Supplementary file 4 — Source Data [file 41467_2020_19632_MOESM4_ESM.zip › Source Data/script_02_consistency_decreases_with_layer_depth/raacampbell-shadedErrorBar/exampleImages/mod-handles.png]

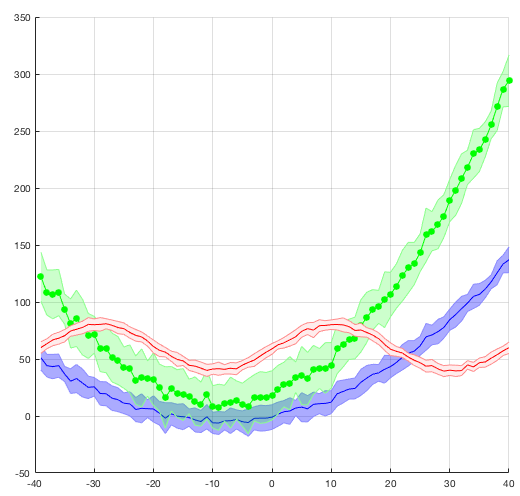

Supplement: Supplementary file 4 — Source Data [file 41467_2020_19632_MOESM4_ESM.zip › Source Data/script_02_consistency_decreases_with_layer_depth/raacampbell-shadedErrorBar/exampleImages/multiple_lines.png]
